# Method and Mechanisms of Soil Stabilization Using Electric Arc Furnace Dust

**DOI:** 10.1038/srep46676

**Published:** 2017-04-28

**Authors:** Omar S. Baghabra Al-Amoudi, Abdullah A. Al-Homidy, Mohammed Maslehuddin, Tawfik A. Saleh

**Affiliations:** 1 Department of Civil and Environmental Engineering, King Fahd University of Petroleum and Minerals, Dhahran, Saudi Arabia; 2 Department of Civil Engineering, Najran University, Najran, Saudi Arabia; 3 Research Institute, King Fahd University of Petroleum and Minerals, Dhahran, Saudi Arabia; 4 Department of Chemistry, King Fahd University of Petroleum and Minerals, Dhahran, Saudi Arabia

## Abstract

This paper reports the method and mechanism for improving the strength of marl and desert sand utilizing electric arc furnace dust (EAFD), an industrial by-product, in lieu of cement or lime. EAFD was used in conjunction with a small quantity (2%) of cement. The mechanical properties and durability characteristics of marl and sand mixed with 2% cement plus 5-, 10-, 20- or 30%-EAFD, by weight of the soil, were evaluated. The soil-cement-EAFD mixtures were used to determine their unconfined compressive strength (UCS), soaked California Bearing Ratio (CBR) and durability. The risk of leaching of toxic heavy metals, such as lead and cadmium, from the stabilized soils to the groundwater was also investigated. The mechanisms of stabilization of the selected soils due to the use of EAFD along with a small quantity of cement are also elucidated. The usage of 20 to 30% EAFD with 2% cement was noted to considerably improve the mechanical properties and durability of both marl and sand.

The increasing volume of industrial waste necessitates the development of economical and efficient methods of their disposal. One of the avenues of the use of waste products is their usage in construction of infrastructure, including the stabilization of weak soils. The soil stabilization using industrial waste materials results in technical, environmental and economic benefits.

Up to 10% cement or lime is often used to improve the strength (stabilization) of weak soils. Since these materials (cement and lime) are expensive and their production requires significant energy and they generate a large volume of greenhouse gases during their production, there is an increasing interest in replacing cement or lime with waste materials for soil stabilization.

Electric arc furnace dust (EAFD) is a high-density by-product of steel production. It has been reported that approximately 15 to 20 kg of EAFD is generated per ton of steel produced^1^,^2^. Approximately 45% of the 1,414 million tons of steel that is produced annually worldwide^3^ is generated via the electric arc furnace method^4^. Therefore, approximately 12 million tons of EAFD is generated annually and disposed of in landfills, creating environmental problems, such as increasing volume of landfill waste, possibility of polluting the groundwater, etc. On the other hand, because of its high fineness, EAFD can be used to improve the mechanical properties of weak soils, such as sand, clay and marl.

There are four main types of soils: marl, sand, clay and salt-crusted sand (called sabkha in some regions)^5^. The latter two types of soils are problematic, in that they swell on exposure to water, and thus, are not often used in the construction of highway embankments. Consequently, marl and sand are the most widely used in the construction of highway embankments. Marl is calcareous in nature and its behaviour is affected by its mineral composition^5^,^6^,^7^,^8^,^9^, density and moisture content, as well as the post-depositional conditions^7^,^9^; thus, it exhibits variable engineering properties. Furthermore, marl is sensitive to inundation, in other words, it loses its strength on exposure to excessive moisture^10^. Despite these drawbacks, marl is the most widely used soil in the construction of highway embankments in many parts of the world due to its easy availability and low cost of processing^5^,^6^,^7^,^8^,^9^,^10^.

Sand is predominantly available in many parts of the world. The two main types of sand – beach sand and desert sand – are both aeolian in nature^11^, that is, their unconsolidated surficial sediments are wind-blown. Most of the desert sand is granular and its behaviour is primarily related to its gradation^12^,^13^,^14^. Further, it is often difficult to compact it as a fill material. Therefore, it cannot be used as a construction material in its natural state and, as such must be stabilized through chemical or mechanical techniques^15^.

About 10% cement or lime is commonly used to improve the strength of a weak soil (stabilization). However, both lime and cement are costly and they are related with high-energy consumption and greenhouse gas emission. Thus, there is a need to study the possibility of utilizing alternative material, particularly industrial waste materials, for soil stabilization. Such usage will lead to technical, economic and environmental benefits. This paper reports a new method for improving the strength and durability of marl and desert sand utilizing EAFD, an industrial by-product, in lieu of cement or lime. The mechanical properties and durability characteristics of marl and sand mixed with 2% cement and 5, 10, 20 or 30% EAFD, by weight of the soil, were evaluated. The mechanisms of stabilization of the selected soils due to the use of EAFD are also elucidated.

## Experimental Procedure

### Materials

#### Marl

**Figure 1:**
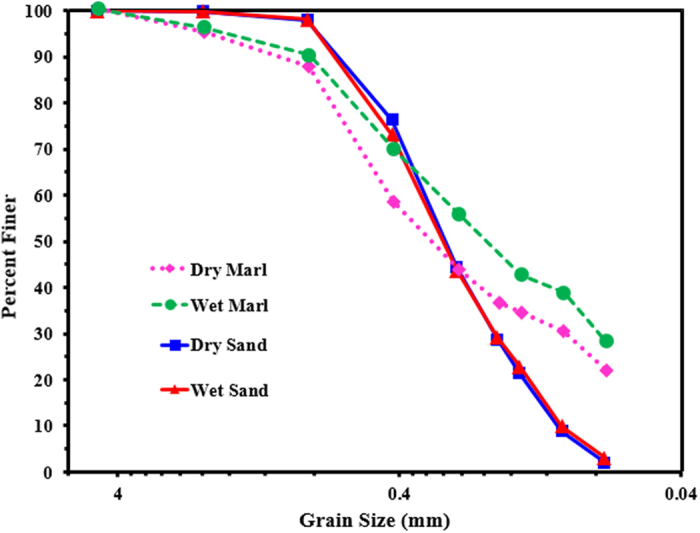
Grain-size distribution of marl and sand.

Marl was sieved to determine its grain-size distribution, as per the procedures outlined in ASTM D422, using dry and wet sieving process. The gradation of the selected marl is depicted in Fig. 1. The specific gravity, determined according to ASTM D854, was 2.69. The Atterberg limits of marl fraction passing through an ASTM #40 sieve were evaluated in accordance with ASTM D423 and D424 and this marl was classified as non-plastic. Based on the grain-size analysis and plasticity^16^, the investigated marl was classified as SM (sandy-marl) according to the Unified Soil Classification System (USCE) and as A-3 (non-plastic) according to the American Association of State Highway Officials (AASHTO) soil classification system.

The mineralogical composition of marl was determined for a soil fraction passing through an ASTM #10 sieve, utilizing a Rigaku Ultima IV X-ray diffractometer. The X-ray peaks indicated that the selected marl contained a high proportion, approximately 62%, of dolomite [CaMg(CO_3_)_2_], 30% quartz (SiO_2_) and 8% calcite (CaCO_3_)^17^,^18^.

#### Sand

The investigated desert sand was sieved in dry and wet conditions according to the procedures outlined in ASTM D422 to determine its grain-size distribution. The results are presented in Fig. 1. The specific gravity of the sand was 2.63. Since the sand was non-plastic in nature, it was classified as A-3, according to the AASHTO soil classification system. The quartz content in the investigated sand was very high (≈100%), as determined by XRD.

#### EAFD

The specific gravity of EAFD, determined in accordance with ASTM D854, was 2.76. Its chemical composition is shown in Table 1. It consisted mostly of iron (probably in the form of iron oxide) and zinc and other heavy metals in small quantities.

### Testing methods

#### Compaction

The optimum moisture content and maximum dry density for each soil-cement-EAFD combinations was determined as per the modified Proctor compaction method (ASTM D1557). The specimens were prepared by thoroughly mixing the dry soil, cement and EAFD for approximately one minute and, after adding water, for another three minutes to obtain a homogenized mixture. Several combinations of density and moisture readings were acquired to determine the maximum dry density and optimum moisture content for each soil-EAFD-cement combination.

#### UCS

The unconfined compressive strength (UCS) was measured in accordance with ASTM D2166. The soil, cement and EAFD mixtures were prepared with the optimum moisture content and were compacted in 100 mm diameter and 200 mm high moulds. The specimens were then wrapped with three layers of plastic sheets to prevent loss of moisture and kept under controlled laboratory conditions (22 ± 3 °C) for seven days. The seven-day curing period represents the practice adopted in the field. Subsequently, they were loaded till failure by applying a compressive load at a deformation rate of 1.25 mm/min. Two soil mixtures were prepared and tested for each mixture and the average UCS values were considered in the evaluation of the results.

#### CBR

The soaked CBR of the treated and untreated soils were evaluated in accordance with ASTM D1883. The moulded and cured specimens were submerged in water for 96 hours (to simulate the fluctuation in the groundwater level) and then measurements were performed.

#### Durability

The durability of the soil-EAFD-cement mixtures was assessed in accordance with ASTM D559. After curing, the specimens were exposed to 12 alternate wet/dry cycles and, subsequently, the weight loss was measured to assess their durability.

## Results and Discussion

### Properties of Marl-Cement-EAFD Mixtures

#### Moisture–Density Relationship

**Figure 2:**
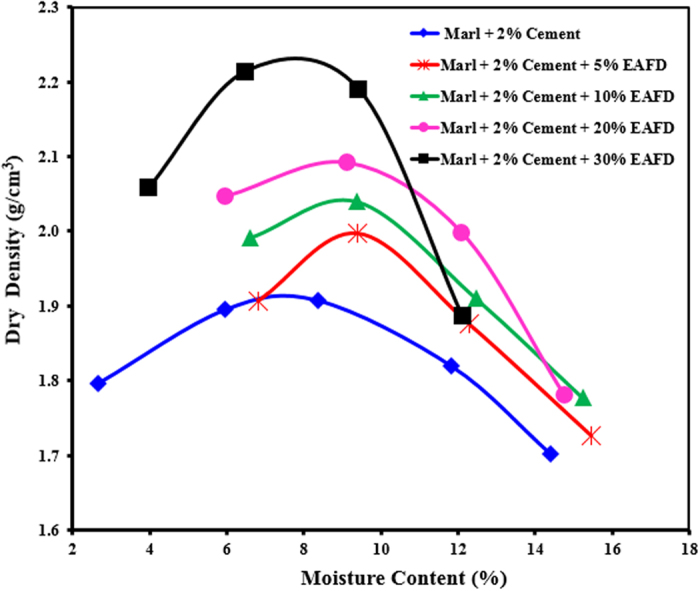
Moisture-dry density relationship for marl-based mixtures.

The compaction tests were conducted on marl mixtures containing 0-, 5-, 10-, 20- or 30%-EAFD to determine the maximum dry density and optimum moisture content. As seen in Fig. 2, the density increased marginally with increasing EAFD content since the specific gravity of EAFD (2.76) is more than that of marl (2.69). The maximum dry density was in the range of 1.90 to 2.23 g/cm^3^, while the optimum moisture content was in the range of 7.6 to 9.2%.

#### UCS

**Figure 3:**
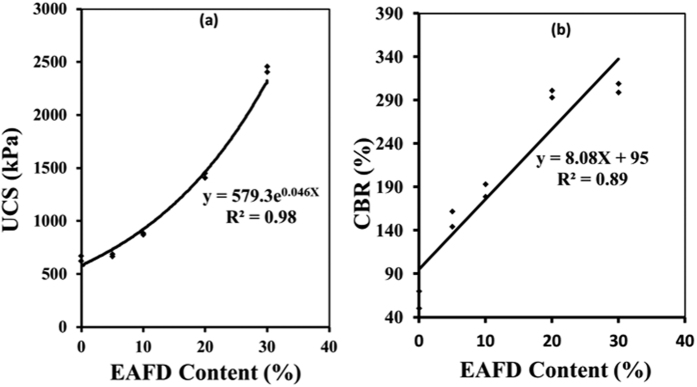
Effect of EAFD content on: (**a**) UCS and (**b**) soaked CBR of the marl-based mixtures.

The UCS of the 0-, 5-, 10-, 20- or 30%-EAFD marl mixtures was in the range of 644 to 2,430 kPa, as shown in Fig. 3a. A significant increase in the UCS was noted at higher EAFD content. There was almost four times increase in the UCS due to the addition of 30% EAFD.

It must be noted that according to the ACI Committee 230 guidelines, the minimum strength requirement for soils to be used in sub-base course in rigid pavements is 1,380 kPa^19^. This requirement is fulfilled by the marl mixture with 20% EAFD. The corresponding requirement for flexible pavement is 1,725 kPa^19^, which is satisfied by the marl mixture with 30% EAFD.

#### Soaked CBR

The soaked CBR of marl increased linearly with the quantity of EAFD, as shown in Fig. 3b. The minimum soaked CBR requirement for a soil to be suitable for application as base-course in both flexible and rigid pavements is 50%^20^,^21^, which is satisfied by all the examined marl-EAFD-cement mixtures.

#### Durability

Moisture and temperature changes can produce wet and dry or freeze and thaw cycles. Hence, stabilized soils should be sufficiently durable to maintain their dimensional stability under these conditions. Consequently, a durability evaluation was conducted on the mixtures that satisfied the UCS requirements (with 20 and 30% EAFD).

The weight loss (indicative of durability) in the marl-cement mixtures with 20 and 30% EAFD was 9 and 8%, respectively. Both of these values are less than the maximum allowable value of 14% specified by the Portland Cement Association for soils classified as SP and 11% required by the US Corps of Engineers for soils with a Plasticity Index (PI) of less than ten^19^.

#### Leachability

The concentration of cadmium and lead in the marl-cement mixture with 20% EAFD was 0.58 and 0.12 mg/l, respectively, and the corresponding values for the 30%-EAFD mixture were 0.67 and 0.17 mg/l, respectively. These values are well below the corresponding allowable values of 1 and 5 mg/l^22^. Therefore, the examined EAFD-stabilized marl mixtures fulfilled the leachability requirements.

### Properties of Sand-Cement-EAFD Mixtures

#### Moisture–Density Relationship

**Figure 4:**
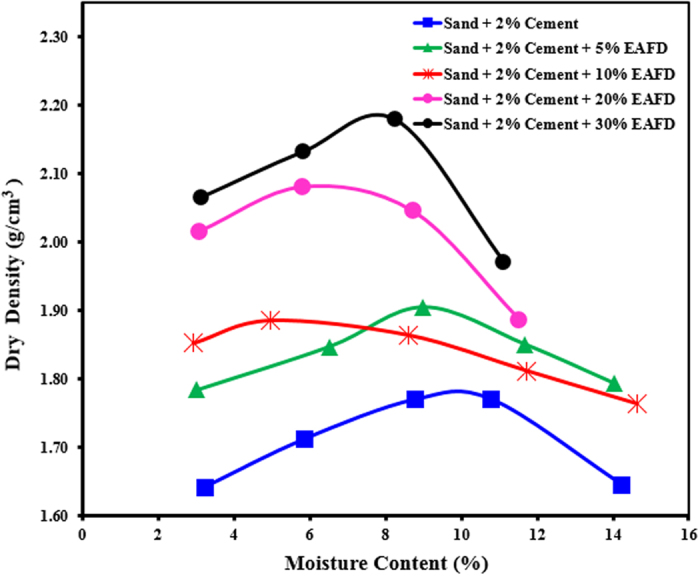
Moisture-dry density relationship for the sand-cement-EAFD mixtures.

The maximum dry density for sand-cement-EAFD (0, 5, 10, 20 and 30%) was in the range of 1.78 to 2.19 g/cm^3^, as shown in Fig. 4. The corresponding optimum moisture content was in the range of 10.0 to 7.8%. The density increased with increasing quantity of EAFD. However, there was not much increase in the optimum moisture content.

#### UCS

**Figure 5:**
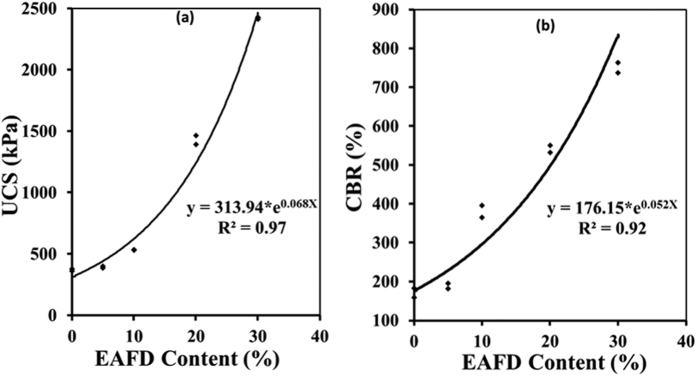
Effect of EAFD content on: (**a**) UCS and (**b**) CBR, of sand with 2% cement (7-days sealed curing).

The UCS of sand-cement-EAFD mixtures was in the range of 369 to 2,419 kPa (Fig. 5a). It may be noted that the mixtures with 20- and 30%-EAFD fulfilled the minimum strength requirements of 1,380 and 1,725 kPa for the sub-base course in rigid and flexible pavements, respectively^19^.

#### Soaked CBR

The soaked CBR of sand-cement-EAFD mixtures was in the range of 171 to 750%, as shown in Fig. 5b. These values are much more than the required value of 50%.

#### Durability

The weight loss of the mixtures with 20- and 30%-EAFD was 9.1 and 7.2%, respectively. Since these two mixtures satisfied the durability requirements, they could be used in the sub-base in both rigid and flexible pavements.

#### Leaching

The concentration of cadmium and lead in the sand-cement mixtures with 20 and 30% EAFD was 0.246 and 0.186 and 0.819 and 0.969 mg/l, respectively. These values are well below the US Environmental Protection Authority (USEPA) restrictions on these metals^22^.

### Mechanisms of Stabilization

The morphology and mineralogical composition of the stabilized marl and sand are mixtures utilized to study the role of EAFD in improving their UCS and soaked CBR of the investigated sand and marl.

### Stabilization of Sand-Cement-EAFD Mixtures

**Figure 6:**
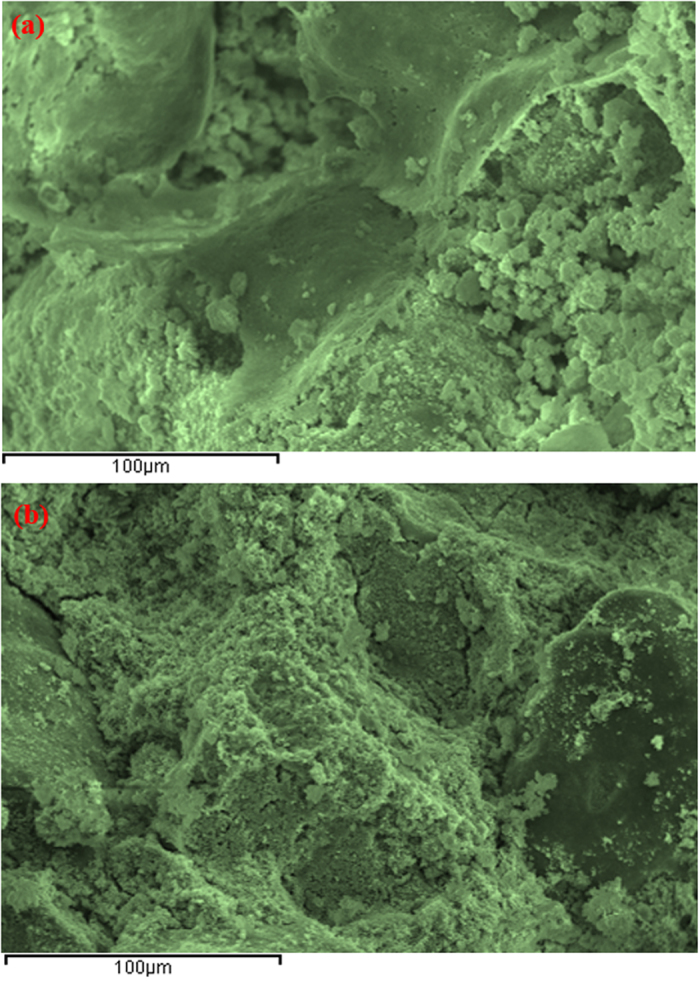
SEM of: (**a**) sand stabilized with 2% cement and (**b**) sand stabilized with 2% cement plus 30% EAFD.

The SEM micrograph of sand-cement plus 20% EAFD, shown in Fig. 6a, indicates the formation of a porous cementing gel, i.e., C-S-H, which develops as a result of the addition of 2% cement to the sand. In contrast, the SEM micrograph of the sand-cement mixture with 30% EAFD, shown in Fig. 6b, shows a dense microstructure.

Compared with the XRD spectra of sand without admixtures (Fig. 6a), which shows mainly quartz, the spectra of the sand-cement-30% EAFD mixture (Fig. 6b) indicates the formation of quartz (SiO_2_, 47%), calcite (CaCO_3_, 2.6%), goethite (FeO(OH), 19.6%) and wustite (FeO, 30.9%). These minerals are formed due to the reaction between sand, cement and EAFD. It may be noted that wustite, in the presence of 2% cement, has good cementing property^17^,^23^,^24^,^25^ and could, therefore, be the main factor causing the observed dense microstructure in the sand-cement-EAFD (30%) mixture, as shown in Fig. 6b.

**Figure 7:**
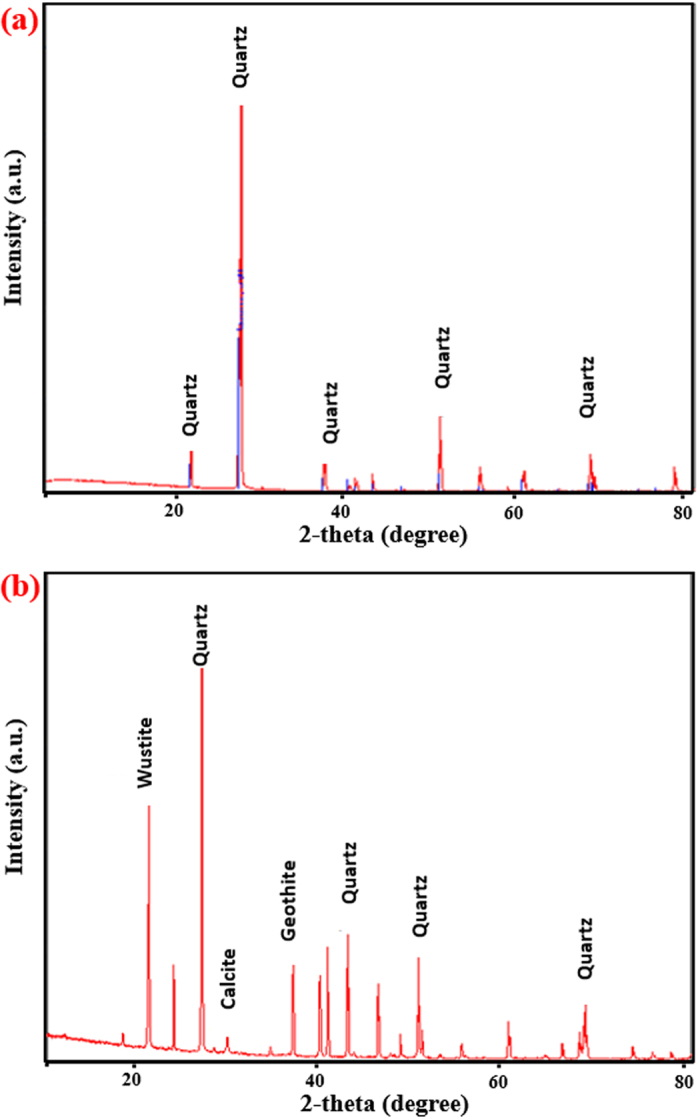
XRD spectra of: (**a**) dune sand and (**b**) dune sand with 2% cement plus 30% EAFD.

**Figure 8:**
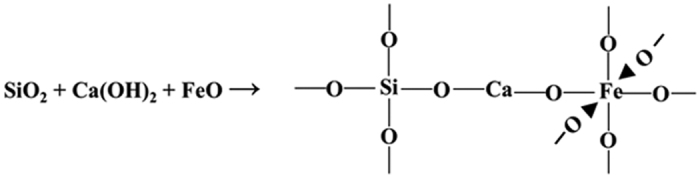
Proposed mechanism of wustite formation in sand stabilized with 2% cement plus EAFD.

Since the electro-negativities of Fe (1.8, Pauling) and Si (1.9 Pauling) are almost the same^26^, the interaction between them is weak. Therefore, in the absence of cement (that is, insufficient Ca), the interaction is retarded and there is no improvement in the UCS. However, improved UCS was measured in sand-cement mixtures with varying quantities of EAFD. This may be attributed to the fact that the low-electronegativity (1.0 Pauling) of Ca in cement facilitates the formation of stronger bonds with both Fe and Si. Under this scenario, the Ca atom is oriented between Fe and Si, thereby generating a binding reaction between silica (quartz from sand), calcium hydroxide (from cement) and iron oxide (wustite and goethite from EAFD), as shown in the XRD pattern in Fig. 7. Therefore, the authors propose the binding sequence, shown in Fig. 8, for the stabilization of sand mixture with 2% cement plus 30% EAFD.

#### Stabilization of Marl-Cement-EAFD Mixtures

**Figure 9:**
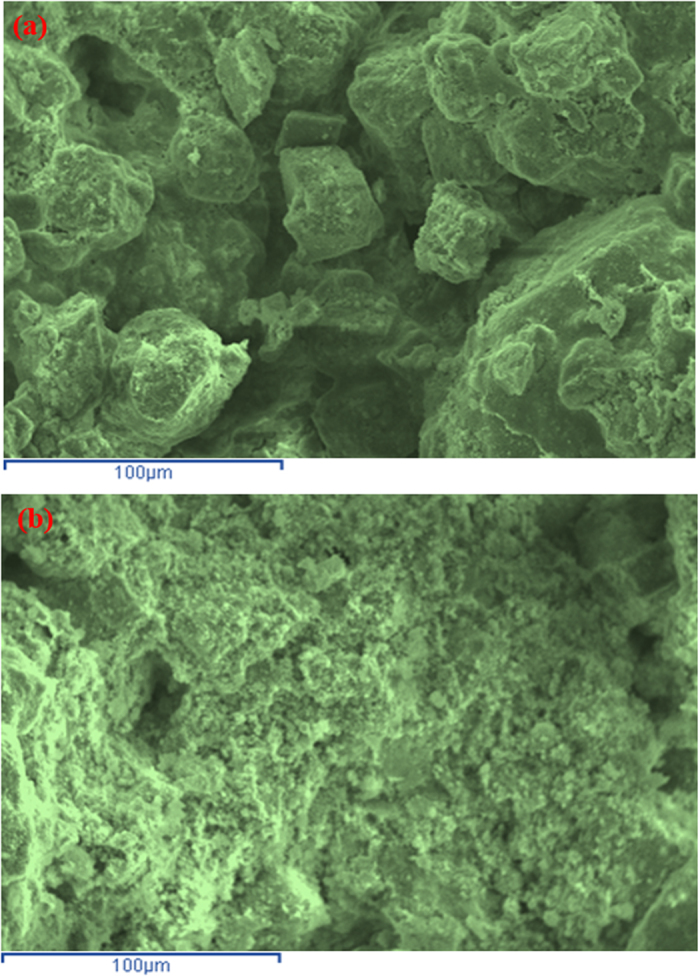
SEM of: (**a**) marl stabilized with 2% cement and (**b**) marl stabilized with 2% cement plus 30% EAFD.

The micrograph of marl stabilized with 2% cement (Fig. 9a) shows a porous microstructure with isolated voids and lack of sufficient cementing gel, suggesting the need for additional stabilizer to create a dense matrix. Comparatively, the SEM of marl stabilized with 2% cement plus 30% EAFD (Fig. 9b) shows a dense morphology, thereby confirming the development of extensive cementitious matrix due to the addition of EAFD.

**Figure 10:**
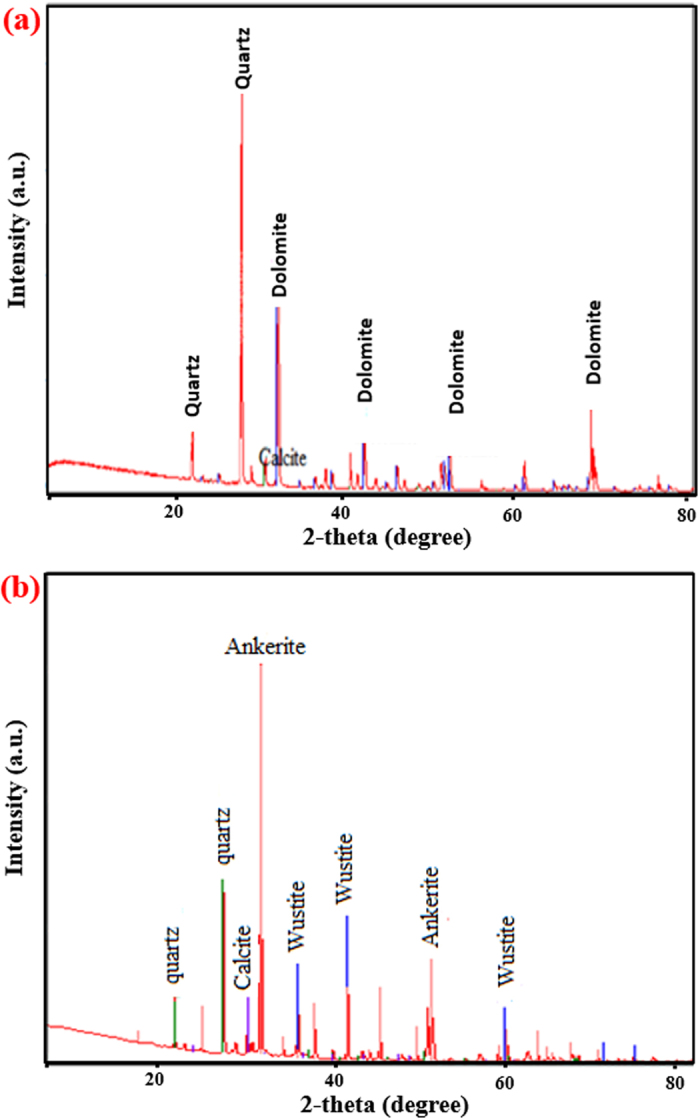
XRD spectra of: (**a**) marl stabilized with 2% cement and (**b**) marl stabilized with 2% cement plus 30% EAFD.

The XRD spectra (Fig. 10) of marl stabilized with 2% cement and 30% EAFD indicate the formation of ankerite ((Ca
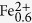
Mg_0.3_
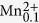
(CO_3_)_2_, 56.6%), wustite (FeO; 20.4%), quartz (SiO_2_; 16.1%) and calcite (CaCO_3_; 6.9%) while these compounds are not noted in marl with cement only. The profuse formation of ankerite and wustite, in the presence of 30% EAFD along with the 2% cement, contributes to the significant increase in the UCS and soaked CBR owing to the excellent cementing properties of these two minerals^17^,^23^,^24^,^25^. These improved properties will certainly lead to stable soil mixtures in highway pavements.

**Figure 11:**
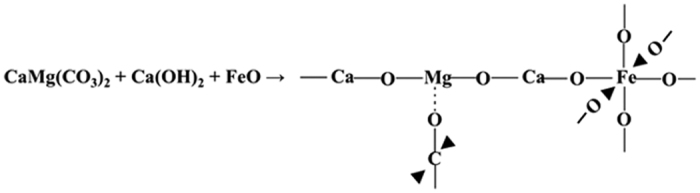
Proposed mechanism of ankerite formation in marl stabilized with cement and EAFD.

The XRD data explain the improvement in the mechanical properties of the marl mixture with cement and EAFD. Quartz and dolomite, in addition to a marginal quantity of calcite, were observed in marl with cement only (Fig. 10a), whereas quartz, wustite and ankerite were present in the XRD spectra of the mixture containing marl, 2% cement plus 30% EAFD (Fig. 10b). As a result of the presence of wustite, the proposed interaction of Si-Ca-Fe discussed earlier may also be partly applicable to the marl mixtures. Based on this assumption, a further interaction between dolomite [CaMg(CO_3_)_2_] (present in the marl), calcium hydroxide (from the cement) and iron oxide (from EAFD) is proposed. This mechanism is based on the electronegativity differences of Mg (1.3 Pauling), Ca (1.0 Pauling) and Fe (1.8 Pauling). Because of its high electronegativity, Fe can form a strong bond with Ca which can then make another bond with Mg to achieve a complete outermost shell with eight electrons. This scenario orients the Ca atom between Mg and Fe, thereby generating a reaction between dolomite, calcium hydroxide and iron oxide. Thus, the interaction illustrated in Fig. 11 is proposed for the formation of ankerite in marl with 2% cement plus 30% EAFD. The improvement in the strength of this mixture can, therefore, be attributed to the binding effect of EAFD through the formation of both wustite and ankerite.

## Conclusion

The findings of this study indicate that marl and sand stabilized with cement and EAFD can be used for the sub-base of rigid and flexible highway embankments. The stabilized soils have proven to be durable and leaching of heavy metals in these mixtures is within the USEPA acceptable limits.

SEM and XRD data were used to propose mechanisms leading to the stabilization of the investigated soils due to the incorporation of EAFD. Specifically, the addition of EAFD to sand-cement mixtures increased the UCS and soaked CBR significantly as a result of the production of wustite. Similarly, an improvement in the properties of marl-cement-EAFD mixtures was ascribed to the formation of wustite and ankerite.

The incorporation of EAFD in weak soils, such as marl and sand, would lead to a reduction in the consumption of cement or lime and the use of EAFD, an industrial waste material. This dual beneficial process would lead to technical, economic and environmental benefits.

## Additional Information

**How to cite this article:** Al-Amoudi, O. S. B. *et al*. Method and Mechanisms of Soil Stabilization Using Electric Arc Furnace Dust. *Sci. Rep.* **7**, 46676; doi: 10.1038/srep46676 (2017).

**Publisher's note:** Springer Nature remains neutral with regard to jurisdictional claims in published maps and institutional affiliations.

## Figures and Tables

**Table 1:** Chemical composition of EAFD.

Element	Weight%
Aluminum	0.70
Calcium	9.39
Cadmium	Negligible
Copper	0.06
Iron	33.6
Potassium	1.7
Magnesium	2.3
Manganese	1.8
Sodium	2.6
Nickel	0.01
Lead	1.31
Phosphorous	0.13
Silicon	2.38
Tin	0.03
Sulphur	0.57
Titanium	0.09
Zinc	10
Oxygen	33.3
